# Tetanus, Cured by Section of the Nerve Supplying the Part

**Published:** 1841-10

**Authors:** 


*Tetanus, cured by section of the nerve supplying the part.* M. Pecchioli has reported, in the Bulletin of Medical Science of Milan, two cases in which he cured tetanus by section of the nerve supplying the part. M. P. says that he had before proposed this operation to a patient, who, however, was unwilling to submit to it and died of the disease. Soon after he met with a well marked case in a young peasant, aged 17. The tetanus was caused by a lacerated wound of the great toe, penetrating the metatarso-phalangean joint; it had continued twenty-four hours. I made, says M. P., an incision eight lines in length, at the point where the saphena passes over the first cuneiform, after having sent a twig to the back of the foot; then I plunged the instrument quite to the bone, to secure the complete division of the nerve. The pain in the leg and foot ceased immediately, and soon after the spasmodic contractions disappeared to return to more. The other case is similar. The editor of the Parisian Medical Gazette commends this operation, and suggests that the section of the nerve could best be made beneath the skin, as in tenotomy. The operation would in that way be a very trifling one, and might be tried in all cases. We shall do so in the first case that offers. *N. Y. Med. Gaz.*

